# Neuromuscular and Functional Adaptations Promoted by Lower Limb Isometric Training with NMES Conditioning Contractions in Older Adults

**DOI:** 10.3390/ijerph23010107

**Published:** 2026-01-13

**Authors:** Jacopo Stafuzza, Federica Gonnelli, Mattia D’Alleva, Maria De Martino, Lara Mari, Simone Zaccaron, Mirco Floreani, Alessio Floreancig, Davide Padovan, Giacomo Ursella, Gabriele Brugnola, Enrico Rejc, Stefano Lazzer

**Affiliations:** 1 Department of Medicine, University of Udine, 33100 Udine, Italy; jacopo.stafuzza@uniud.it (J.S.); federica.gonnelli@uniud.it (F.G.); mattia.dalleva@uniud.it (M.D.); maria.demartino@uniud.it (M.D.M.); mari.lara@spes.uniud.it (L.M.); simone.zaccaron@uniud.it (S.Z.); enrico.rejc@uniud.it (E.R.); 2 School of Sport Sciences, University of Udine, 33100 Udine, Italy; floreancig.alessio@spes.uniud.it (A.F.); padovan.davide@spes.uniud.it (D.P.); ursella.giacomo@spes.uniud.it (G.U.); brugnola.gabriele@spes.uniud.it (G.B.); 3 Aging Research Center, Department of Neurobiology, Care Sciences and Society, Karolinska Institute and Stockholm University, 17165 Stockholm, Sweden; 4 Department of Neurosciences, Biomedicine and Movement Sciences, University of Verona, 37129 Verona, Italy

**Keywords:** electrical stimulation, lower limb, older adults, isometric training

## Abstract

**Highlights:**

**Public health relevance—How does this work relate to a public health issue?**

- The present work addresses the effects of two training modalities on strength, power and functional capacities in older adults. The aforementioned factors are strongly related to the health and functional independence of this population, which is one most in need of public health interventions.

**Public health significance—Why is this work of significance to public health?**

- This work broadens the knowledge related to the implementation of isometric training and neuromuscular electrical stimulation for improving health-related factors in older adults. In this population, physical activity is particularly beneficial to delay and sometimes offset the age-related loss of strength, power and overall functional independence, thus improving quality of life.

**Public health implications—What are the key implications or messages for practitioners, policy makers and/or researchers in public health?**

- The present study shows that a combination of isometric training and neuromuscular electrical stimulation implemented unilaterally improves strength, power and functional capacities in older adults.
- The proposed training intervention also promoted a small but significant cross-education effect. Hence, the proposed approach may also be considered in situations where only one limb can be exercised (i.e., rehabilitation settings).

**Abstract:**

Aging induces sarcopenia and reduces bone mineral density, altering body composition. These modifications contribute to physical decline, increase non-communicable disease risk and increase the likelihood of hospitalization, thereby representing a substantial public health burden. In this study, we assessed the effects of isometric training with neuromuscular electrical stimulation conditioning contractions (ISO-NMES) and dynamic resistance training (DRT) on physical and functional capacities. Moreover, we investigated the impact of ISO-NMES training on the force and power of the trained and untrained leg. Eighteen sessions of ISO-NMES training for knee extensors were performed by 10 older adults (age: 70.1 ± 4.9 years; ISO-NMES group). The DRT group (*n* = 12; age: 70.5 ± 2.8 years) performed 18 sessions of dynamic resistance training at a local fitness center. Maximum voluntary contraction (MVC) and peak power (P) of lower limbs as well as functional capacities assessed with the 5 Sit to Stand, Timed Up and Go and 6 Minutes Walking Tests were examined in both groups before and after the related training protocols. At the end of the training period, only the ISO-NMES group had improved MVC (+30.4%, *p* < 0.001) and bilateral force (ISO-NMES: +6.3%, *p* = 0.032). Moreover, both groups had significantly improved functional capacities. Finally, in the ISO-NMES group, MVC, force and power significantly increased in both legs with a greater effect for MVC in the trained than untrained leg (+30.4 vs. +13.5%, *p* < 0.001). These findings suggest that ISO-NMES training was an effective strategy to improve physical and functional capacities in older adults. Thus, it could be considered as a potential intervention, particularly when the mobility to perform physical training is limited.

## 1. Introduction

With aging, the human body undergoes numerous adaptations including modification of body composition, decreased bone density and loss of muscle quality and mass (sarcopenia) [1]. Such changes can decrease the quality of life and increase the risk of developing concurrent pathologies (i.e., cardiovascular or metabolic diseases), which could lead to hospitalization [2].

Elderly individuals experience loss of motor neurons, accompanied by remodeling of surviving motor units, changes in muscle fiber type composition and a decrease in neuromuscular drive [3,4]. The decline in strength, impaired motor coordination and disruption in excitation–contraction coupling contribute to a reduction in overall physical performance [2,5]. Furthermore, a specific reduction in the size of type II muscle fibers has been observed in older adults, with these fibers being approximately 10–40% smaller compared to those in younger individuals [6]. This reduction is critical, as type II fiber loss negatively affects muscle power more rapidly than muscle strength [7]. However, although aging is characterized by a decline in both muscle quality and quantity, skeletal muscle in older adults remains highly responsive to resistance training stimuli.

Resistance training is recognized as the most effective non-pharmacological intervention to increase strength and muscle mass in humans [8,9]. Dynamic resistance training (DRT) is characterized by force exertion against an external load through alternating phases of muscle shortening and lengthening [10]. A collective view of the literature highlights the importance of mechanical load for muscle hypertrophy [11]. Moreover, there is substantial evidence suggesting that mechanical load-induced alterations in whole-muscle cross-sectional area can be driven by an increase in fascicle diameter and pennation angle [12,13]. Therefore, it is important to select appropriate training strategies to optimize physical and functional capacities.

Resistance training benefits older adults by preserving bone density, increasing muscle mass, enhancing muscle strength and potentially reducing the risk of falls and related injuries [14,15]. Furthermore, a strength-focused training intervention performed 2 to 3 times per week (20 to 30 min per session) can contribute to lowering the risk of cardiovascular disorder, diabetes and osteoporosis [16,17]. Multi-joint exercises such as rowing, shoulder press, chest press, squat, deadlift and others targeting multiple muscle groups simultaneously can be particularly effective to enhance muscle strength and support independence and daily activities [18]. Indeed, increasing muscle strength is associated with increased walking performance, improved ability to rise from a sitting position and overall mobility [19].

Isometric resistance training was shown to be an effective alternative option for increasing strength, hypertrophy and dynamic performances in both athletes and older adults [20,21]. Moreover, promising results come from the combination of isometric training and neuromuscular electrical stimulation (NMES), making it an interesting approach to enhance strength training [22]. Previous work from our group also demonstrated that using NMES to condition the neuromuscular system was more effective than voluntary contractions for improving the rate of torque development of knee extensors [23]. Post-activation potentiation was conceivably contributed to such positive adaptation. Overall, isometric training with NMES can be considered a potentially valuable training strategy to adopt when impaired mobility or other limitations do not allow more dynamic training modalities.

Therefore, the aim of the present study was to compare the effects of isometric training with NMES conditioning contractions (ISO-NMES) versus dynamic resistance training (DRT) on physical and functional capacities in older adults. Our hypothesis was that ISO-NMES and DRT would elicit similar effects on physical and functional capacities.

## 2. Materials and Methods

### 2.1. Subjects

Twenty-two healthy older adults (11 males and 11 females) agreed to participate in this study. Participants had a full medical history and physical examination to test their eligibility for the study. Inclusion criteria were as follows: participant age of between 65 and 75 years; being able to walk independently for at least 1 km; and absence of mobility limitations; neurological, musculoskeletal and metabolic diseases; and/or frailty linked to bone health problems. Exclusion criteria were as follows: having any cardiovascular or neurological conditions; having one or more implanted electrical devices; having orthopedic impairments; and using psychotropic drugs or any medication that could influence strength and power expression. All subjects were sedentary [24] (data not reported) and did not participate in any other physical activity protocols immediately prior to and during the study. Participants were assigned to the ISO-NMES group (ISO-NMES, 6 males and 4 females) or the DRT group (DRT, 5 males and 7 females) based on their availability to travel to the training facilities according to the training schedules. The trainers that supervised the training interventions were not informed about the aims of the study. The study was conducted in accordance with the principles outlined in the Declaration of Helsinki (1975, revised in 2013) and was approved by the Institutional Review Boards of the University of Udine (Italy) (038/IRB DAME_2021, approved on 7 June 2021). Written informed consent was obtained from all subjects involved in the study.

### 2.2. Intervention Protocol

The ISO-NMES group was asked to complete 18 training sessions, with 6 weeks of ISO-NMES training (3 sessions per week) of the knee extensors of one leg (i.e., trained leg), identified as the dominant leg. The dominant leg was considered as the leg used to kick a ball [25]. The DRT group performed dynamic resistance training for a total of 18 training sessions, with 2 sessions per week for 9 weeks. Before and after the training intervention, anthropometric measurements, maximal voluntary isometric contraction, explosive power of the lower limbs and physical performance through functional tests were assessed.

### 2.3. Training Intervention—ISO-NMES Group

**Figure 1:**
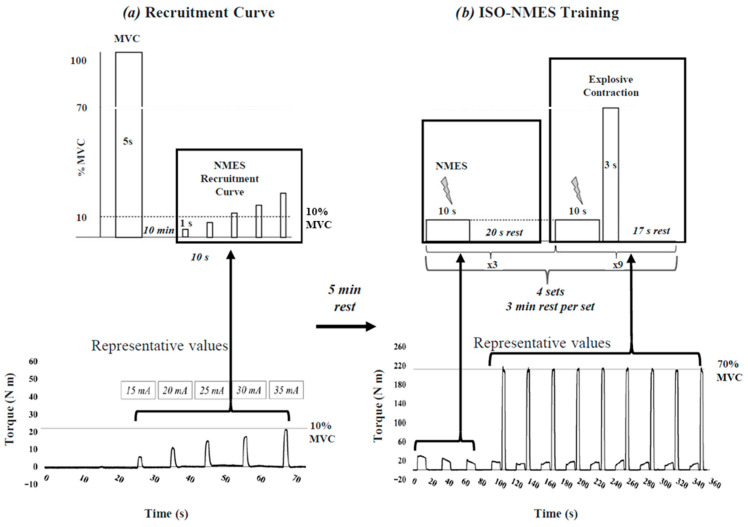
Schematic representation of the experimental protocol for the ISO-NMES group. (**a**) Recruitment curve protocol; (**b**) ISO-NMES training protocol.

The training protocol for the ISO-NMES group consisted of a warm-up, the NMES recruitment curve, the ISO-NMES training and a final cool down phase. Each training session lasted approximately 60 min. The training protocol for the ISO-NMES group was conducted as follows: First, NMES pads (size: 5 cm × 10 cm; Axelgaard Manufacturing Co., Ltd., Fallbrook, CA, USA) were positioned over the quadricep muscle belly with the distal portion placed at 50% and 10% of the distance between the anterior superior iliac spine and the superior margin of the patella, for proximal and distal pads, respectively [26]. Then, subjects sat on the isometric dynamometer and performed an initial warm up consisting of 20 to 30 contractions (4 s each), at self-selected and increasing intensity. After a 3 min rest period, participants performed a maximal isometric knee extension lasting 5 s to determine MVC (Figure 1a). After 10 min from the MVC attempt, an electrical stimulator (Digitimer DS7A, Welwyn Garden City, Hertfordshire, UK; Maximal Voltage 400V) was used to deliver a 1 s monophasic positive rectangular waveform with 1000 µs pulse width at a constant frequency and voltage of 100 Hz and 400 V, respectively. Stimulation trains were delivered to the muscle every 10 s, starting at an amplitude of 5 mA and increasing by 5 mA for every subsequent stimulation until a minimum torque equal to 10% of MVC was elicited, and either the participant requested to stop because of discomfort or the recruitment curve reached a plateau [27] (Figure 1a). This protocol was selected as it was previously demonstrated to be effective for enhancing muscle contractile properties in healthy adults [23]. After 5 min of rest, the isometric training protocol began. It consisted of 4 sets separated by 3 min of rest in between sets. Each set included 3 stimulations of 10 s each, separated by 20 s. These were followed by 9 cycles of stimulation and explosive isometric contractions at 70% of MVC, with each contraction lasting 3 s and each cycle separated by 17 s of rest (Figure 1b; see [23] for further details). Finally, the cool down phase consisted of pedaling on a stationary bike at 80rpm for 5 min at a self-selected intensity. All the training sessions were supervised by a trained sport scientist.

### 2.4. Training Intervention—DRT Group

The protocol for the DRT group consisted of a combination of body weight, elastic band and light dumbbell exercises for approximately 60 min, 2 times per week for 9 weeks at a fitness center supervised by a personal trainer. The first part of the session (5 to 10 min) was a warm-up that targeted the major joints. The subsequent main part of the session consisted of 40 min of exercises, equally divided for the upper and lower body. More specifically, the 4 exercises for the upper body were standing row, standing chest press, arm curls and lateral raises; the 4 exercises for the lower body were box squat, seated leg extension, glute bridge and calf raise. After the completion of this part of the session, the participants performed 5–10 min of cooldown, which included stretching the main muscle groups. The exercise volume for the main part consisted of 2–3 sets per 10–15 repetitions each. Loads were selected by the trainer to induce an individual rate of perceived muscular exertion equal to 4 points according to the RPE scale (Borg CR10 [28]).

### 2.5. Anthropometric Characteristics

Body mass (BM) was measured to the nearest 0.1 kg with a manual weighing scale (Seca 709, Hamburg, Germany) with the subject dressed only in light underwear and no shoes. Stature was measured to the nearest 0.5 cm on a standardized wall-mounted height board. The body mass index (BMI) was calculated as BM (kg) stature-2 (m).

### 2.6. Maximal Voluntary Contractions

Participants performed MVCs of the knee extensors of the dominant lower limb while sitting on the isometric dynamometer. After the warm-up and a 3 min rest period, participants were asked to perform a maximal isometric knee extension for approximately 6 s. Three MVC attempts were performed, separated by a 5 min rest, and the highest peak force was considered for further analysis. All data were collected as a force output and then transformed in torque data during off-line analysis. To calculate the torque value in each subject, force values were multiplied by the force lever arm which was the distance between the center of the knee joint and the 5 cm proximal to the superior malleoli of the ankle where the center of the force cell (AM C3, Laumas Elettronica, Mantua, Italy; Sensitivity: 2.2 mv/V ± 10%) was placed. Torque was recorded by custom LabVIEW software 2021 (National Instrument Inc., Austin, TX, USA) and sampled at 1 kHz. LabChart 8 (ADInstruments, Dunedin, New Zealand) was used to low-pass filter all torque data at 10 Hz and for the subsequent analysis.

Additionally, in the ISO-NMES group alone, MVC of the contralateral knee extensors was also tested to assess any potential cross-education effect of the unilateral ISO-NMES training.

### 2.7. Maximal Explosive Power

Dynamic explosive power of lower limbs was assessed by means of the explosive ergometer (EXER), as previously described [29]. More specifically, the EXER device was formed by a metal frame that supported a rail inclined at 20° from the ground. A seat was attached to the rail and was free to slide on a carriage. At the starting position, the seat faced two force plates (LAUMAS PA 300, Parma, Italy) and was also attached to a tachometer (LIKA SGI, Vicenza, Italy). Force and velocity analog outputs were sampled at 1000 Hz using a data acquisition system (MP100; BIOPAC Systems, Inc., Goleta, CA, USA). During the test, the subject was seated, secured by a safety harness fastened around the shoulders and abdomen, with his arms on the handlebars, and was instructed to maximally accelerate himself, allowing the carriage to slide on the rail. In such a way, it was possible to record the force reaction performed by the feet on the force plate and the peak velocity reached by the system. Two mechanical blocks were used to set the distance between the seat and the force platforms, so that the knee angle at rest was 110°. The blocks also prevented any countermovement and, consequently, any recovery of elastic energy during the pushing phase. The tests were performed by asking the subject to push with both legs. After a brief familiarization, explosive efforts were repeated 3 times with 2 min of rest in between attempts.

The instantaneous bilateral power (P_BIL_) was calculated from the product of instantaneous bilateral force (F_BIL_) and velocity (v_BIL_) values; the attempt with the greatest peak power was then refrained for further analysis. For statistical analysis, the peak power was normalized by body weight.

Furthermore, in the ISO-NMES group alone, force (F), velocity (v) and power (P) were also tested unilaterally, for both legs, to assess any potential cross-education effect of the unilateral ISO-NMES training.

### 2.8. Functional Performance Evaluations

Functional performance was evaluated using the 5 Sit to Stand Test (5STS), the Timed Up and Go Test (TUG) and the 6 Minutes Walking Test (6MWT). In the 5STS Test, the participant was asked to stand up and sit down five times in a row from a seated position on a chair without using their arms for assistance. The total time to complete 5 repetitions was recorded and used for further analysis. Then, during the TUG test, the participant was asked to stand from a chair, walk for 3 m, turn around a cone and walk back to sit on the chair as in the starting position. The time to complete the test was used for the analysis.

During the 6MWT, each participant was instructed to cover as much distance as possible at a self-selected speed in 6 min. The total distance covered was used for further analysis.

### 2.9. Statistical Analysis

All results are expressed as mean and standard deviation (SD). Normal distribution of the data was tested using the Kolmogorov–Smirnov test. Sphericity (homogeneity of covariance) was verified by Mauchly’s test. When the assumption of sphericity was not met, the significance of the F-ratios was adjusted according to the Greenhouse–Geisser procedure. To assess differences in the data between groups after the completion of the training period, statistical analysis was performed in JASP 0.95.4 (Amsterdam, The Netherlands) using a Linear Mixed Model with significance set at *p* ≤ 0.05, with group and time as fixed factors, including their interactions and baseline values as covariates. The significance of fixed effects was assessed using Fisher’s *F*-statistics, with degrees of freedom estimated using the Satterthwaite approximation. Further investigations were conducted using Bonferroni-corrected post hoc comparisons. Changes in the trained and untrained leg were investigated within the ISO-NMES group using a two-way repeated-measure ANOVA with *p* ≤ 0.05. Significant differences were further investigated using Bonferroni-corrected post hoc comparison. In addition, effect size (ES) was calculated using Cohen’s *d* (0 < *d* < 0.20, *small*; 0.20 < *d* < 0.50, *medium*; 0.50 < *d*, *large*). A sensitivity analysis was conducted using G*Power 3.1 (Heinrich-Heine-Universität Düsseldorf, Germany) to estimate the sensitivity of the study for the primary outcome only (Maximal Voluntary Contraction, MVC). Assuming a medium effect size (*d* = 0.40), a correlation of 0.5 between repeated measures and a pre–post between-group design, the analysis indicated that a total sample size of 16 participants would provide a statistical power of 0.94.

## 3. Results

All participants enrolled in the study completed the assessment and training sessions. No dropouts after the start of the training period were observed.

### 3.1. Anthropometrics

Baseline values of age, stature, body mass and BMI were not significantly different between the two groups.

### 3.2. MVC and Power

Baseline values for MVC and power were not significantly different between groups (Table 1). At the end of the training period, we observed an increase in MVC for the ISO-NMES group only (+30.4%, *p* < 0.001, F = 52.9; Table 1). In addition, F_BIL_ only increased significantly in the ISO-NMES group (F_BIL_: +6.3%, *p* = 0.032, F = 14.4). Conversely, v_BIL_ and P_BIL_ showed a non-significant positive trend (v_BIL_: +5,6%, *p* = 0.053, F = 4.1; P_BIL_: +8.3%, *p* = 0.059, F = 5.6) for the ISO-NMES group only.

### 3.3. Functional Tests

Baseline values for 5STS, TUG and 6MWT test were not significantly different between groups (Table 1). At the end of the training period, 5STS improved significantly in the ISO-NMES and DRT groups (−15.8 and −15.1%, *p* < 0.001, F = 39.5, respectively). In addition, TUG improved significantly in the ISO-NMES and DRT groups (−9.9 and −9.3%, *p* < 0.001, F = 21.4, respectively). Finally, 6MWT increased significantly more in the ISO-NMES than DRT group (+9.4 vs. +5.8%, *p* = 0.006, F = 4.9) (Table 1).

### 3.4. MVC and Power in Trained and Untrained Leg in ISO-NMES Group

In the ISO-NMES group, MVC increased significantly in both legs with a medium effect for the trained leg (+30.4 vs. +13.5%, *p* < 0.001, ES = 0.52; Table 2). Finally, small and similar improvements were observed for force and power values in the trained and untrained leg (F: +5.8% and +3.3%, *p* = 0.013, ES = 0.14; P: +7.9% and +7.1%, *p* = 0.016, ES = 0.19, respectively, Table 2).

## 4. Discussion

The main findings of the present study were as follows: (i) isometric training combined with NMES significantly increased MVC and bilateral force in the ISO-NMES group; (ii) both groups (ISO-NMES and DRT) improved performance in the 5STS, TUG and 6MWT tests; (iii) finally, in the ISO-NMES group, MVC increased significantly in both legs with greater effect in the trained versus untrained leg.

As hypothesized, ISO-NMES and DRT similarly improved functional performance tests (5STS, TUG, 6MWT). Conversely, our findings showed the specific advantages of ISO-NMES on maximal isometric strength of knee extensors (MVC). The training intervention proposed in the ISO-NMES group implemented an NMES conditioning contraction protocol with the main goal of promoting a post-activation performance enhancement to be exploited during the voluntary isometric knee extension training. The ISO-NMES group demonstrated significant increases in MVC and force and marginal gains in power, which is consistent with previous findings [30]. Although older adults are generally less responsive to post-activation potentiation, this reduced responsiveness is likely due to age-related alterations in excitation–contraction coupling mechanisms and atrophy-related factors [31,32]. Atrophy-related factors refer to the gradual loss of muscle mass that occurs with aging. This loss can contribute to decreased muscle function and increased vulnerability to injury. No significant differences in MVC were found in the DRT group; this could be partially explained by the difference in training frequency and intensity between the two groups despite the same total number of training sessions. Training 2–3 times a week has been shown to provide an optimal training stimulus in older adults [14]. Also, training intensity represents a key determinant of strength gains, with previous evidence indicating that the greatest improvements were typically observed at intensities of 70–80% of 1RM [33]. In the present study, only the ISO-NMES group trained within this intensity range, whereas the DRT group trained at a more moderate training intensity due to the nature of the intervention. Differences in training intensity could have contributed to the observed differences in MVC gains between the two groups.

Both the ISO-NMES and DRT groups showed similar improvements in functional capacities, as evidenced by enhanced performance in the 5STS, TUG and 6MWT tests. Improvements in the 5STS Test may be associated with the increased lower limb strength, which is essential for performing daily movements [34,35]. Improvements in TUG suggest that both groups had improved mobility and functional balance [36]. Interestingly, the ISO-NMES group improved more in the 6MWT. Improvement in this test, which assesses aerobic and functional capacities [37], suggests a general improvement in aerobic and lower limb capacities. This could be related to the reduction in energy cost of walking [38] and may promote an improved capacity to perform daily tasks [39]. The similar magnitude of improvement in functional outcomes may be due primarily to the structured and supervised nature of the training interventions rather than the specific training modality. Several studies have shown that structured aerobic, resistance or combined training enhances physical performance in older adults [40]. Regular resistance training enables older adults to outperform their sedentary peers and maintain function comparable to younger individuals [41]. While strength and hypertrophy are key determinants of functional performance [42], muscle mass and architecture were not measured in this study. This precludes a comprehensive mechanistic understanding of the structural adaptations underlying the functional improvements herein reported. However, previous studies with similar training durations suggest that the observed strength improvements could be primarily mediated by neural adaptations [43,44]. Moreover, combined NMES and isometric training has previously been shown to improve strength, balance, mobility and functional outcomes, such as the 6MWT and TUG tests [22,45]. However, in the absence of a non-exercise control group, it is not possible to draw definitive conclusions regarding the impact of ISO-NMES training, despite statistically significant improvements.

The improvements in 6MWT and 5STS met established Minimal Clinically Important Differences (MCIDs) for community-dwelling older adults. The ISO-NMES (+54m) and DRT (+31m) groups exceeded the 20–30m threshold for meaningful change in the 6MWT [46]. For the 5STS, both groups moved further from the 12 s fall-risk cut-off identified by Tiedemann and collaborators [47].

Furthermore, we also found improvements in MVC for the untrained leg in the ISO-NMES group. This increase could be explained by a cross-education effect, which involves complex neural mechanisms. Hortobágyi et al. 1999 [48] investigated this phenomenon by applying voluntary and NMES-evoked isometric and eccentric contraction training on one leg only. They found that the strength of the untrained leg (i.e., contralateral) improved significantly, despite being relaxed during the entire intervention with NMES-evoked contraction training [48]. Also, Cattagni et al. 2018 found MVC improvements in the contralateral (i.e., not trained) leg after implementing a low-intensity NMES protocol [49]. In a recent review, Calvert and Carson [50] investigated the main mechanisms that may mediate the expression of the cross-education effect, with insights into age-related changes. The authors concluded that, although the primary pathway may involve the primary motor cortex 1, the underlying adaptations are more likely to involve other brain regions. Furthermore, age-related variations across the lifespan are unlikely to significantly alter the state of corticospinal projections that terminate on motor pools innervating the untrained leg.

Considering the findings of the present study, isometric strength training with NMES conditioning should be kept in consideration for those scenarios in which movement is restricted (i.e., hospitalization, bed rest and similar), even if it could only be performed unilaterally (i.e., because of a fracture and subsequent cast placed on the contralateral side). Future research directions could proceed along two main directions: (i) add an isometric-only group to isolate the contribution of NMES to improvements in strength, power and functional performance; (ii) conduct follow-up assessment to determine whether the gains are retained over time.

### Limitations

A limitation of this study was the absence of an isometric-only training group, which prevented us from isolating the specific contribution of NMES conditioning contractions to the strength, power and functional gains observed in the ISO-NMES group. Furthermore, the lack of a non-exercise control group precluded us from establishing clear causal training effects. Although both interventions followed standard resistance training guidelines and the overall training volume was similar, training frequency and intensity differed between groups. The relatively small sample size (*n* = 22) may have affected the generalization of the results and limited the statistical power to detect meaningful effects for outcomes other than MVC. In addition, participants were assigned arbitrarily to the two groups based on their availability. Finally, due to logistics constraints, no long-term follow-up was conducted; thus, the interpretability of the findings herein was limited to short-term adaptations, and no conclusions could be drawn regarding the retention of training effects.

## 5. Conclusions

Eighteen sessions of a combination of isometric training with NMES-elicited conditioning contractions appeared to be effective in increasing knee extensors’ muscle strength and power in elderly adults. These improvements also affected the untrained leg, showing a potential cross-education effect. Additionally, changes in muscle performance were accompanied by improvements in functional test outcomes. Further studies with larger samples are needed to confirm these results and to evaluate the efficacy of adding this training modality to more comprehensive exercise protocols or rehabilitation settings for older adults.

## Figures and Tables

**Table 1:** Anthropometric characteristics and functional test values of participants before (PRE) and after (POST) intervention.

	DRT (*n* = 12)						ISO-NMES (*n* = 10)									
	PRE			POST			PRE			POST			T	G	T × G	F
Age (y)	70.1	±	4.9				70.5	±	2.8					0.796		
Stature (m)	1.66	±	0.10				1.67	±	0.10					0.898		
Body mass (kg)	68.9	±	13.4				67.9	±	12.8					0.821		
BMI (kg/m^2^)	25.0	±	3.4				24.4	±	2.5					0.591		
MVC (Nm)	143.3	±	36.4	140.7	±	37.3	163.4	±	54.1	213.1	±	66.2 *	0.001	0.001	0.001	52.9
F_BIL_ (N)	925.1	±	234	968.6	±	237.7	1014.8	±	372.5	1082.1	±	362.9 *	0.001	0.376	0.422	14.4
v_BIL_ (m/s)	1.5	±	0.3	1.5	±	0.3	1.7	±	0.3	1.8	±	0.3 *	0.040	0.015	0.049	4.1
P_BIL_ (W)	1178.4	±	495.1	1196.1	±	476.0	1458.7	±	627.9	1591.2	±	642.8	0.025	0.059	0.081	5.6
5STS (s)	8.9	±	2.3	7.5	±	1.7 *	11.6	±	2.8	9.7	±	2.4 *	0.001	0.417	0.345	39.5
TUG (s)	5.5	±	1.0	5.0	±	0.9 *	6.9	±	1.2	6.2	±	0.8 *	0.001	0.169	0.528	21.4
6MWT (m)	543.8	±	88.6	575.7	±	81.1 *	579.8	±	76.1	634.7	±	76.7 *	0.001	0.014	0.031	4.9

*: *p* < 0.05: difference between PRE vs. POST; F: Fisher’s F-statistics; BMI: body mass index; MVC: Maximal Voluntary Contraction; F_BIL_: bilateral force; v_BIL_: bilateral velocity; P_BIL_: bilateral power; 5STS: 5 Sit to Stand Test; TUG: Timed Up and Go Test; 6MWT: 6 Minutes Walking Test.

**Table 2:** MVC, force, velocity and power in trained (T) and untrained (UT) legs of the ISO-NMES group observed PRE and POST intervention.

	Trained Leg						Untrained Leg									
	PRE			POST			PRE			POST			T	G	T × G	ES
MVC (Nm)	163.4	±	54.1	213.1	±	66.2 *	159.9	±	39.8	181.5	±	60.4 *	0.001	0.012	0.016	0.52
F (N)	662.9	±	198.6	703.6	±	190.9 *	696.5	±	207.1	721.3	±	193.5 *	0.013	0.077	0.695	0.14
v (m/s)	1.3	±	0.2	1.4	±	0.3	1.4	±	0.2	1.4	±	0.3	0.065	0.234	0.178	0.26
P (W)	813.8	±	306.2	879.4	±	338.5 *	843.3	±	313.1	903.4	±	330.7 *	0.016	0.090	0.766	0.19

*: *p* < 0.05: difference between PRE vs. POST; ES: effect size; MVC: Maximal Voluntary Contraction; F: force; v: velocity; P: power.

## Data Availability

The data presented in this study are available on request from the corresponding author.

## Abbreviations

The following abbreviations are used in this manuscript:

ANOVA	Analysis of variance
BIL	Bilateral leg
BM	Body mass
BMI	Body mass index
DRT	Dynamic resistance training
EXER	Explosive ergometer
F	Force
ISO-NMES	Isometric training with NMES conditioning contractions
MVC	Maximum voluntary contraction
NMES	Neuromuscular electrical stimulation
P	Power
T	Trained leg
TUG	Timed Up and Go Test
UT	Untrained leg
v	Velocity
6MWT	6 Minutes Walking Test
5STS	5 Sit to Stand Test
